# Dynamic Digital Radiography (DDR) for Pulmonary Blood Flow Assessment After Lung Transplantation: Insights From Two Case Studies

**DOI:** 10.7759/cureus.82485

**Published:** 2025-04-18

**Authors:** Satoshi Komatsu, Tomoyuki Nakamura, Yasushi Hoshikawa, Yasushi Matsuda, Osamu Nishida

**Affiliations:** 1 Anesthesiology and Critical Care Medicine, Fujita Health University, Toyoake, JPN; 2 Thoracic Surgery, Fujita Health University, Toyoake, JPN

**Keywords:** complication, diaphragmatic function, dynamic digital radiography, low-dose imaging, lung transplant, pulmonary embolism (pe), pulmonary perfusion scintigraphy, thrombosis

## Abstract

Dynamic digital radiography (DDR) is an innovative imaging modality that enables real-time evaluation of dynamic processes by combining sequential images. DDR can assess pulmonary blood flow by analyzing variations in black and white signal intensities within the lung field. This case series evaluates the utility and limitations of DDR for monitoring pulmonary blood flow and thrombosis in two patients following lung transplantation. DDR findings were compared with those of pulmonary perfusion scintigraphy (PPS), the current gold standard for assessing regional pulmonary perfusion distribution. Case 1 is a male in his 40s who underwent a single (left) lung transplantation. Postoperative DDR indicated pulmonary perfusion distributions between the autologous (right) and transplanted (left) lungs of 46.89% vs. 53.11% at 3.5 months and 38.02% vs. 61.98% at six months. However, PPS indicated markedly different distributions: 15.78% vs. 84.22% at two months and 19.34% vs. 80.66% at six months. Case 2 is a male in his 40s who underwent bilateral sequential lung transplantation. DDR indicated pulmonary perfusion distributions between the right and left transplanted lungs of 51.10% vs. 48.90% at two months and 48.48% vs. 51.52% at six months. In this case, PPS findings were in close agreement: 54.82% vs. 45.18% at two months and 48.70% vs. 51.30% at six months. Contrast-enhanced CT confirmed the absence of thrombosis and flow abnormalities. DDR imaging in lung transplant patients offers a low-radiation and convenient approach for monitoring respiratory function and assessing complications. However, our findings indicate that DDR assessments of pulmonary blood flow do not consistently match those of PPS, the current reference standard. Caution and further investigations regarding the use of DDR are needed, including in the context of thrombosis evaluation.

## Introduction

Dynamic digital radiography (DDR) (AeroDR Trauma & X-ray; Konica Minolta, Chiyoda City, Japan) is an innovative imaging technique that enables real-time evaluation of respiratory and circulatory dynamics, offering insights that have been challenging to obtain with conventional radiographic methods. DDR was commercially introduced in 2018. By combining high-speed digital detectors with advanced image processing, DDR provides continuous imaging capabilities that have shown promise in various clinical applications [1,2]. DDR assesses blood flow by monitoring changes in the X-ray signal and can effectively visualize pulmonary circulation when subjects briefly hold their breath.

In lung transplantation, a critical treatment for end-stage lung diseases, postoperative complications such as diaphragmatic nerve damage and pulmonary thrombosis can significantly impact patient outcomes [3-6]. DDR holds potential for identifying these complications and monitoring pulmonary blood flow distribution pre- and post-transplantation.

Pulmonary perfusion scintigraphy (PPS) is the current standard for evaluating pulmonary blood flow and diagnosing conditions such as pulmonary embolism and chronic pulmonary artery thrombosis [7-9]. However, DDR may serve as a complementary tool, particularly in the post-lung transplantation setting where the pulmonary artery provides the sole blood supply to the transplanted lung, and thrombosis poses a significant risk to graft viability.

Stationary DDR technology has been used in most DDR studies, while portable DDR devices remain underexplored despite their advantages in various medical settings. Portable DDR, unlike PPS, abandons the need to transport the patient to a different facility, which is highly advantageous when extracorporeal circulation is being performed, as in this case.

This study aims to evaluate the utility of portable DDR in assessing pulmonary blood flow and detecting thrombosis in two post-lung transplantation cases - one with single-lung and the other with bilateral sequential transplantation - while exploring its potential as a low-radiation, bedside alternative to PPS.

## Case presentation

Case 1

A male in his 40s, 172.5 cm tall and weighing 67.6 kg, was receiving home oxygen therapy for idiopathic interstitial pneumonia. Following identification of a brain-dead donor, he underwent a single (left) lung transplantation supported by veno-arterial extracorporeal membrane oxygenation (V-A ECMO). V-A ECMO was not required after the implantation of the organ and was successfully weaned intraoperatively. The patient was admitted to the ICU while still intubated. Extubation occurred on postoperative day five, and the patient left the ICU on postoperative day 23. He was discharged on postoperative day 84. As a note, the cases described in this study were not particularly severe, but relatively long ICU periods are common policy in Japan, and we opted for even further prolonged close monitoring to learn from these cases.

**Figure 1:**
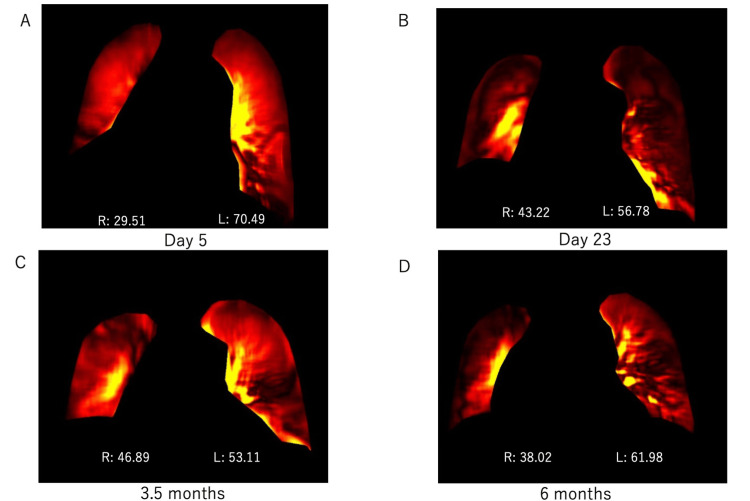
Pulmonary blood flow ratios between the autologous and transplanted lungs in Case 1, at different times post-surgery: dynamic digital radiography (DDR) results. A: Day 5, B: Day 23, C: 3.5 months, D: 6 months, R: right, L: left The number indicates the percentage, which is 100% including the left and right sides.

Portable DDR was conducted on postoperative days five and 23 and 3.5 and six months to evaluate pulmonary blood flow. The pulmonary blood flow distributions between the autologous (right) and transplanted (left) lungs were as follows: postoperative day five, 29.51% vs. 70.49%; day 23, 43.22% vs. 56.78%; 3.5 months, 46.89% vs. 53.11%; and six months, 38.02% vs. 61.98% (Figure 1).

**Figure 2:**
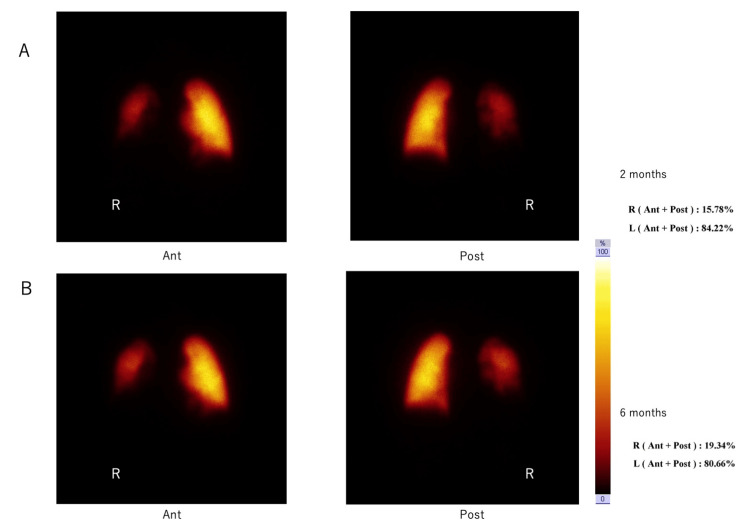
Pulmonary blood flow ratios between the autologous and transplanted lungs in Case 1, at different times post-surgery: pulmonary perfusion scintigraphy (PPS) results. A: 2 months, B: 6 months, R: right, Ant: Image taken from the front, Post: Image taken from the back The number indicates the percentage, which is 100% including the left and right sides.

However, compared to DDR, PPS performed at two and six months showed very different distributions (right vs left): 15.78% vs 84.22% and 19.34% vs 80.66%, respectively (Figure 2).

Contrast-enhanced CT was performed at two and six months postoperatively, and no thrombus was detected.

Case 2

A male in his 40s, 156.4 cm tall and weighing 53.6 kg, was hospitalized for bilateral pneumothoraces associated with fibrotic nonspecific interstitial pneumonia (NSIP) while on the waiting list for lung transplantation. After a brain-dead donor was identified, he underwent bilateral sequential transplantation supported by V-A ECMO. V-A ECMO was not required post-implantation of the organ and was successfully weaned intraoperatively. The patient was admitted to the ICU while still intubated. Extubation occurred on postoperative day five, and the patient left the ICU on day 16. He was discharged on day 177.

**Figure 3:**
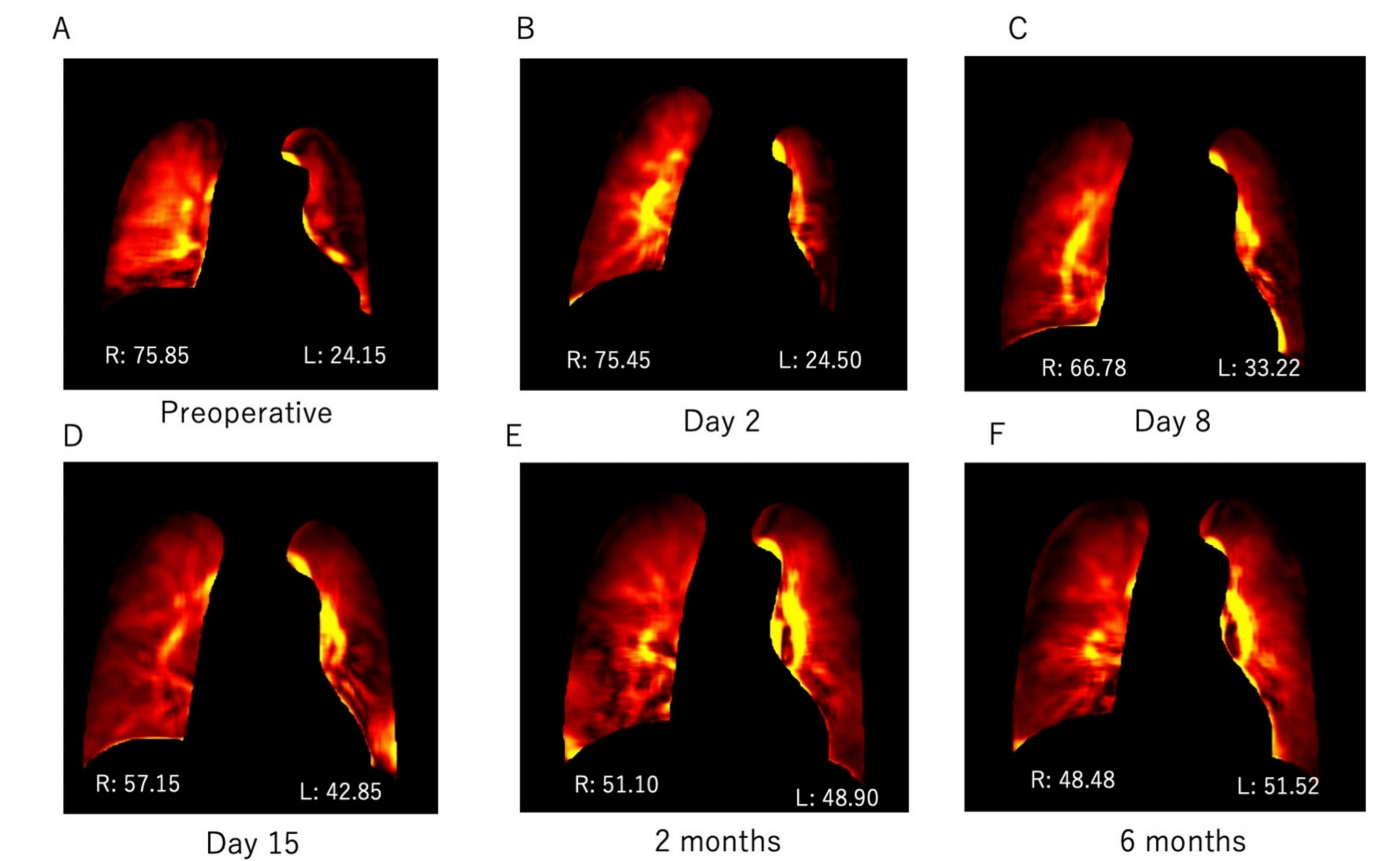
Pulmonary blood flow ratios between the transplanted right and left lungs in Case 2, pre-operatively and at different times post-surgery: dynamic digital radiography (DDR) results. A: Pre-operative, B: Day 2, C: Day 8, D: Day 15, E: 2 months, F: 6 months, R: right, L: left The number indicates the percentage, which is 100% including the left and right sides.

Portable DDR demonstrated changes in pulmonary blood flow ratios between the transplanted right and left lungs as follows: pre-operatively, 75.85% vs. 24.15%; post-operative day two, 75.45% vs. 24.50%; day eight, 66.78% vs. 33.22%; day 15, 57.15% vs. 42.85%; at two months, 51.10% vs. 48.90%; and at six months, 48.48% vs. 51.52% (Figure 3).

**Figure 4:**
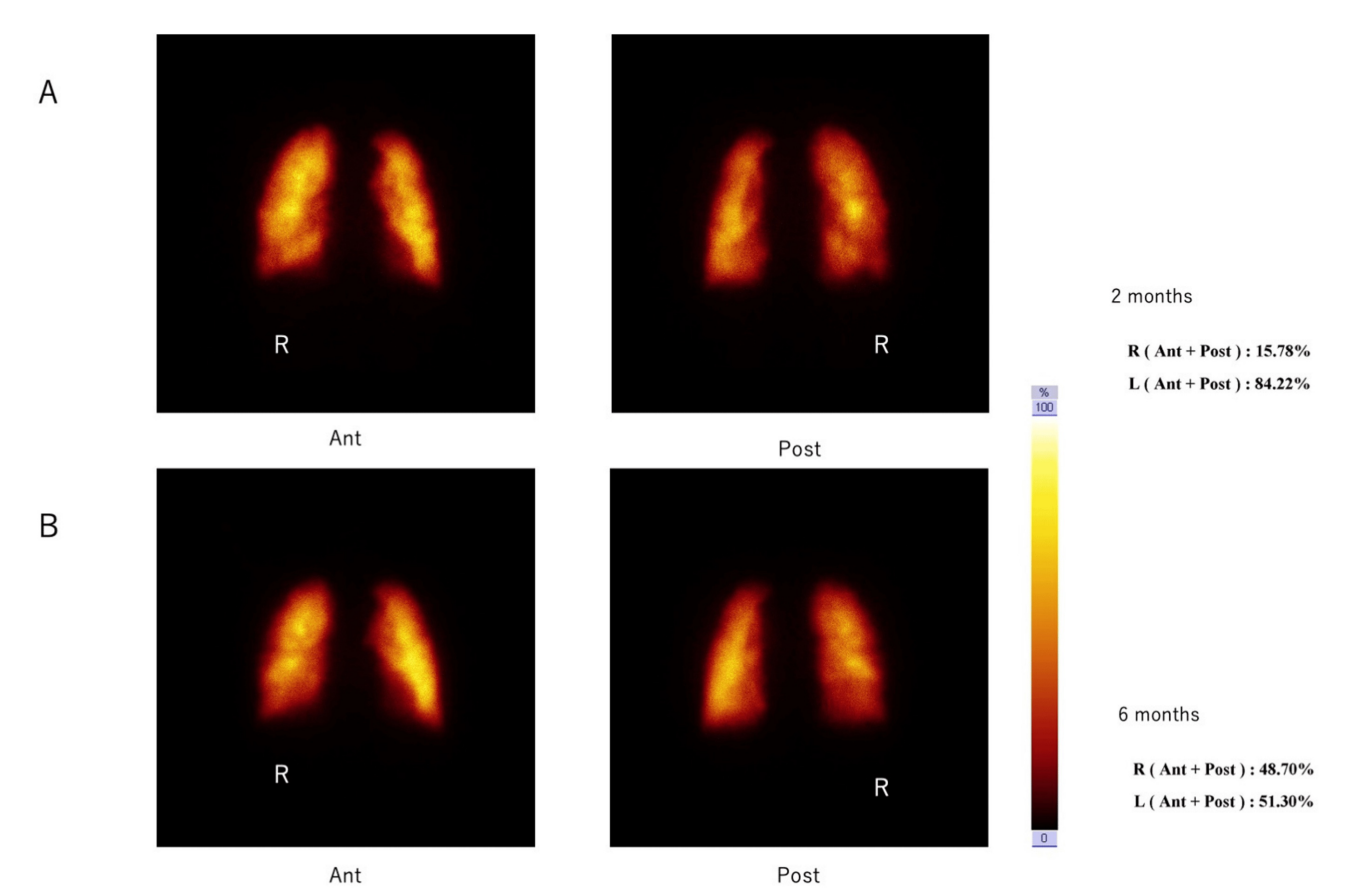
Pulmonary blood flow ratios between the transplanted right and left lungs in Case 2, pre-operatively and at different times post-surgery: pulmonary perfusion scintigraphy (PPS) results. A: 2 months, B: 6 months, R: right, Ant: Image taken from the front, Post: Image taken from the back The number indicates the percentage, which is 100% including the left and right sides.

PPS data were consistent with the DDR data and showed the following right versus left distributions: at transplant enrollment in 2015, 79.8% vs. 20.2%; at two months post-operatively, 54.82% vs. 45.18%, and at six months, 48.70% vs. 51.30% (Figure 4).

The patient was extubated on the fifth post-operative day. Respiratory status did not worsen before or after extubation. On the same day, laboratory results showed elevated fibrin degradation products (FDP: 47.2 µg/mL; reference range < 5 µg/mL) and D-dimer levels (24.5 µg/mL; reference range ≦1 µg/mL), prompting contrast-enhanced CT - solely as a careful precaution - to evaluate for thrombosis. However, no thrombus was detected. Further CT scans at two and six months post-operatively confirmed the absence of thrombotic complications.

## Discussion

**Figure 5:**
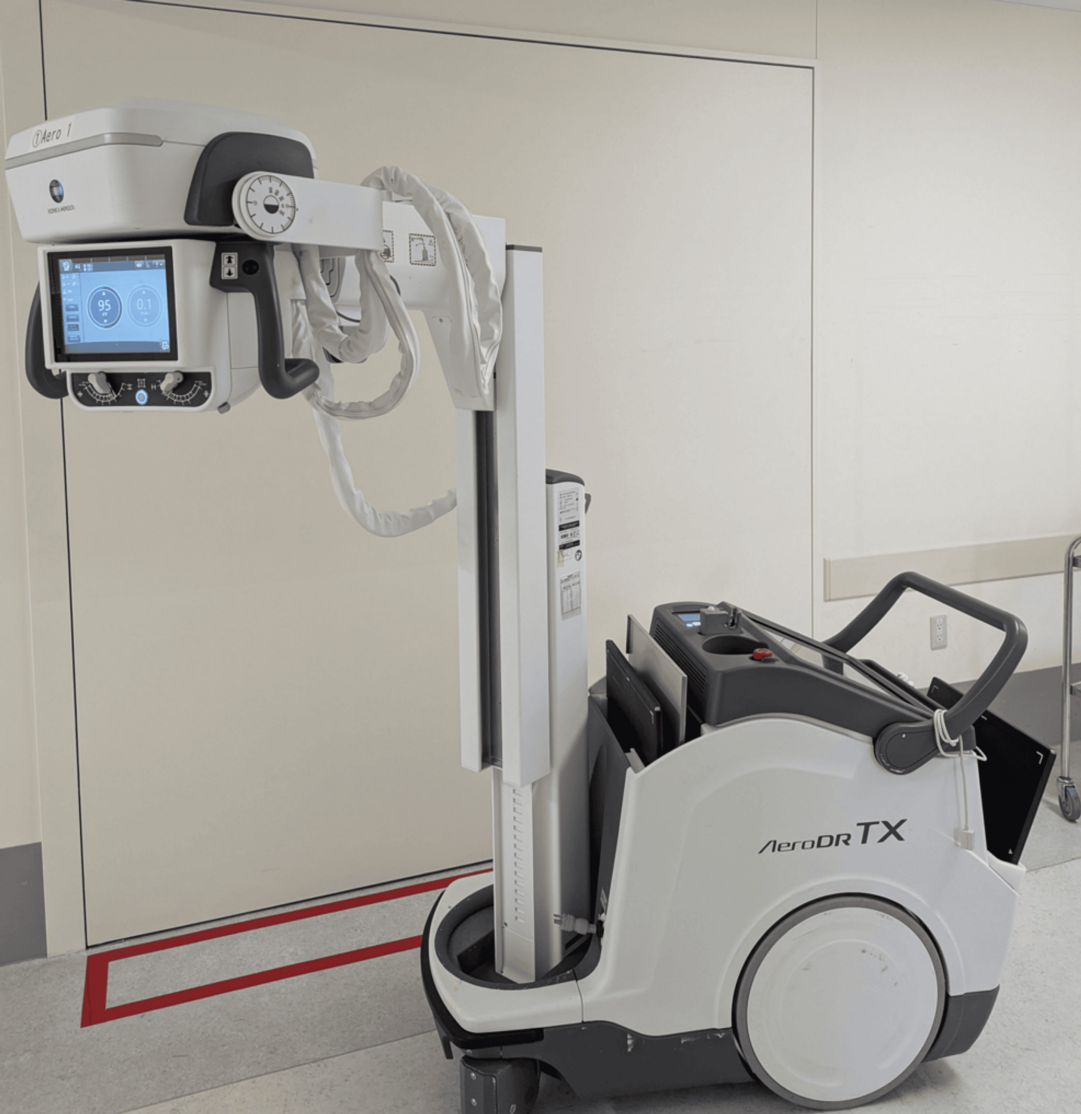
Photograph of the mobile dynamic digital radiography equipment (AeroDR Trauma & X-ray; Konica Minolta, Chiyoda City, Japan), which has a portable detector panel.

**Figure 6:**
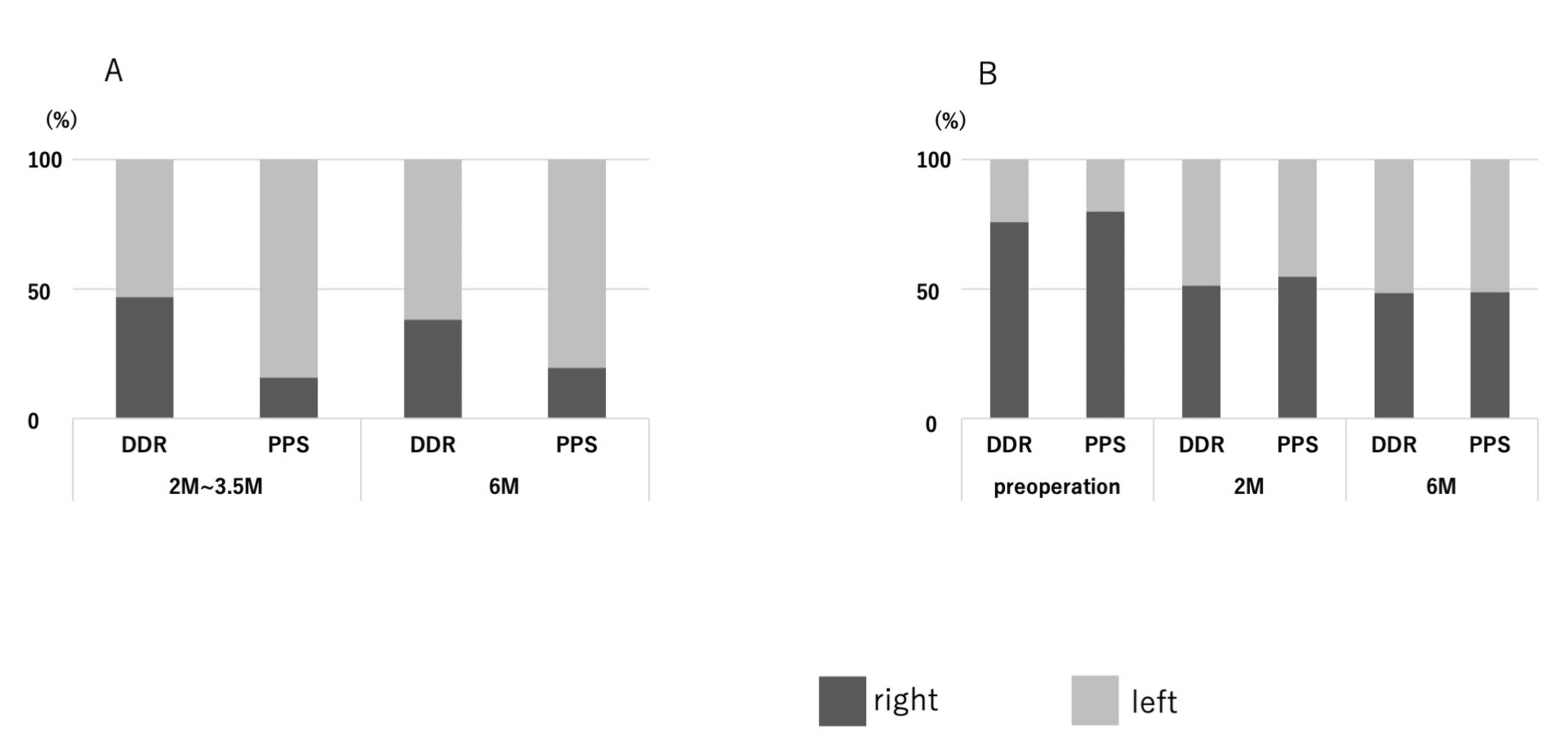
Comparison of pulmonary blood flow ratios obtained at similar time points in Case 1 (A) and Case 2 (B) using DDR or PPS. The Y-axis represents the proportions of the right lung (dark gray) and left lung (light gray) contributing to the total (100%) of the observed flow. The X-axis represents times: pre-operatively and 2, 3.5, and 6 months post-surgery (see Figures 1-4). DDR: Dynamic digital radiography; PPS: Pulmonary perfusion scintigraphy

DDR and PPS results on pulmonary blood flow do not consistently agree. In this study, portable DDR (Figure 5) imaging was utilized to assess pulmonary blood flow in lung transplant patients, and the results were compared to PPS. In Case 1, there was a marked discrepancy between DDR and PPS results, whereas, in Case 2, there was close agreement between the two modalities. PPS results supported that in Case 2, DDR effectively captured the temporal dynamic changes in blood flow distribution between right and left lungs, with preoperatively a dominant role for the right lung, and similar blood flows in both transplanted lungs from two to six months post-operatively (Figure 6).

These findings underscore both the potential and limitations of DDR in clinical practice. PPS uses radiolabeled agents that accumulate in pulmonary capillaries, providing precise imaging of blood flow distribution. On the other hand, DDR detects changes in X-ray signals during breath-holding to assess blood flow, which may lead to inaccuracies in specific regions, such as those posterior to the heart or during suboptimal breath-holding. Such limitations may have contributed to the discrepancies observed in Case 1. We also speculate that, for unknown reasons, DDR allows a proper comparison between two autologous lungs or two transplanted lungs (as in Case 2), but not between an autologous and a transplanted lung (as in Case 1). Regardless, the concordance between DDR and PPS in Case 2 suggests that DDR has the potential to complement PPS, especially in cases where bedside imaging is advantageous. It will be necessary to more precisely determine the conditions under which DDR produces meaningful results.

Clinical significance of DDR in lung transplantation 

DDR offers several advantages over traditional imaging modalities. Its portable design and low radiation dose make it particularly suited for frequent monitoring in lung transplant recipients, who often require repeated imaging [10]. DDR tends to expose patients to only approximately 1/20th of the radiation dose required for CT, making it safer, especially for long-term follow-up [8]. Unlike PPS, which necessitates radiolabeled agents and specialized facilities, DDR can be performed at the bedside with minimal logistical challenges. We envision that, if DDR were to become similarly reliable and informative on pulmonary blood flow as PPS, DDR may even be used to capture blood flow changes during the acute phase of lung transplantation. An additional advantage of DDR over PPS or CT is its ability to provide insights into diaphragmatic function (Video 1), a critical factor in post-transplant recovery. 

**Video 1:** This is a dynamic image captured using DDR. It was taken six months after Case 1, in the supine position. Dynamic digital radiography (DDR) sequence obtained six months post-operatively in Case 1, with the patient in the supine position. The video illustrates pulmonary perfusion dynamics during tidal breathing, highlighting differences between the transplanted and native lungs. Movement of the diaphragm is also clearly visualized throughout the respiratory cycle.

Previous studies have demonstrated that DDR can detect blood flow defects associated with thrombosis without the need for contrast agents [8,9]. This capability is particularly relevant, as approximately 6% of lung transplant recipients experience post-operative pulmonary embolism (PE), and early detection is essential for preventing graft or patient loss [6]. Although thrombotic complications were not identified in the present cases, DDR’s ability to assess pulmonary blood flow dynamically and noninvasively suggests its utility in early diagnosis and monitoring of thrombosis.

Technical challenges and limitations

Despite its promise, DDR has several limitations that must be addressed. First, DDR does not provide three-dimensional imaging, which limits its ability to capture certain pathologies. Second, as a relatively new technology, DDR's analytical methods are not yet standardized, which may impact the consistency and reliability of its results. Third, DDR’s dynamic imaging is not completely real time, introducing a slight time lag of typically a few seconds for the imaging itself and up to minutes for software calculations, and the duration of imaging sessions is constrained by patient cooperation, especially regarding breath-holding. Finally, DDR is not yet widely available, restricting its accessibility in many clinical settings and slowing the generation of new data.

Future directions

Standardizing DDR protocols for pulmonary blood flow assessment and further refining its analytical methods will be crucial for its broader adoption. Expanding its use in clinical studies with larger patient cohorts could validate its efficacy and reliability. If these challenges are addressed, DDR has the potential to become a standard tool for pulmonary evaluation, particularly in lung transplantation, where early detection of complications is critical for improving patient outcomes.

## Conclusions

Portable DDR imaging offers a low-dose radiation exposure, an accessible method for monitoring respiratory function and pulmonary blood flow in lung transplant recipients. However, the case-dependent discrepancies between DDR and PPS data in this study highlight that DDR's reliability for assessing pulmonary blood flow varies across clinical scenarios. The critical factors contributing to these differences have yet to be identified. Thus, DDR should currently be employed cautiously as a complementary tool rather than a standalone diagnostic method. Its clinical utility and standardization will require further validation through prospective observational studies involving larger numbers of both single lung and bilateral sequential transplantation cases.
